# Supplementary material for: Mangrove-Derived Endophytic Bacteria Enhance Growth, Yield, and Stress Resilience in Rice
Source: Int J Mol Sci. 2025 Sep 24;26(19):9317. doi: 10.3390/ijms26199317 (PMC12525508; doi:10.3390/ijms26199317)
Supplement: Supplementary file 1 [file ijms-26-09317-s001.zip › ijms-3870230-supplementary.pdf]

Supplementary Figures

Figure S1: Screen for potential PGPBs of the isolated mangrove bacterial collection in *A. thaliana*.

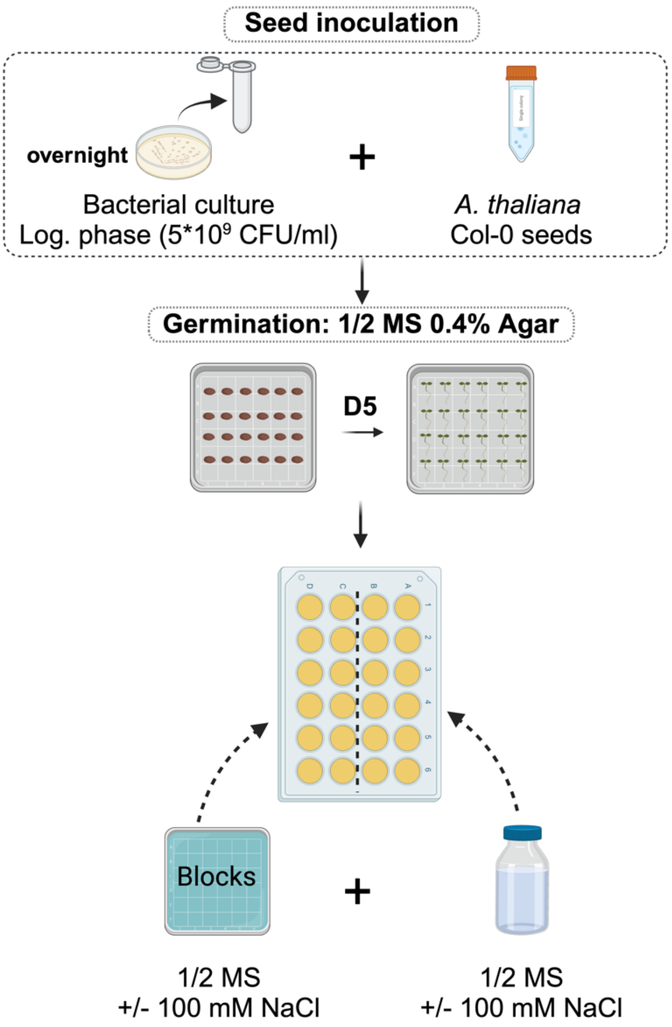

Figure S2: Workflow of the hydroponic and greenhouse experiments using modified Hoagland nutrient solution.

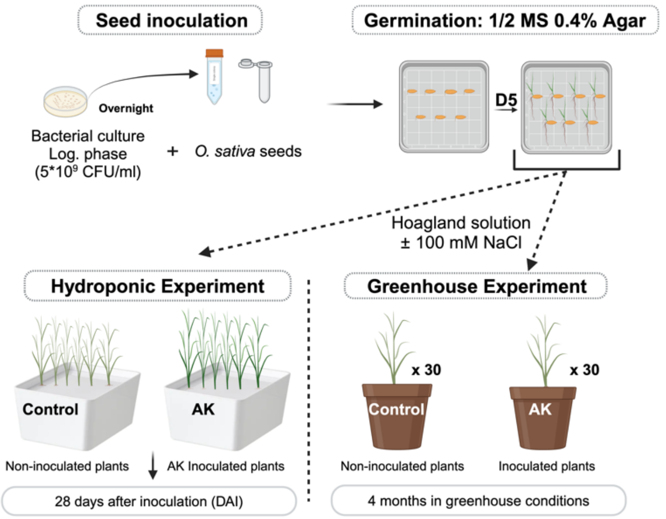

Figure S3: Shoot RNA sequencing data. DEGs of shoots of rice plants grown under normal and saline hydroponic conditions stress treated with AK164, AK171, or MOCK inoculated. Number of DEGs (log 2 > 1, P < 0.01) in rice shoots colonized with AK164, AK171, or MOCK under normal (0 mM NaCl) and saline (100 mM NaCl) hydroponic conditions.

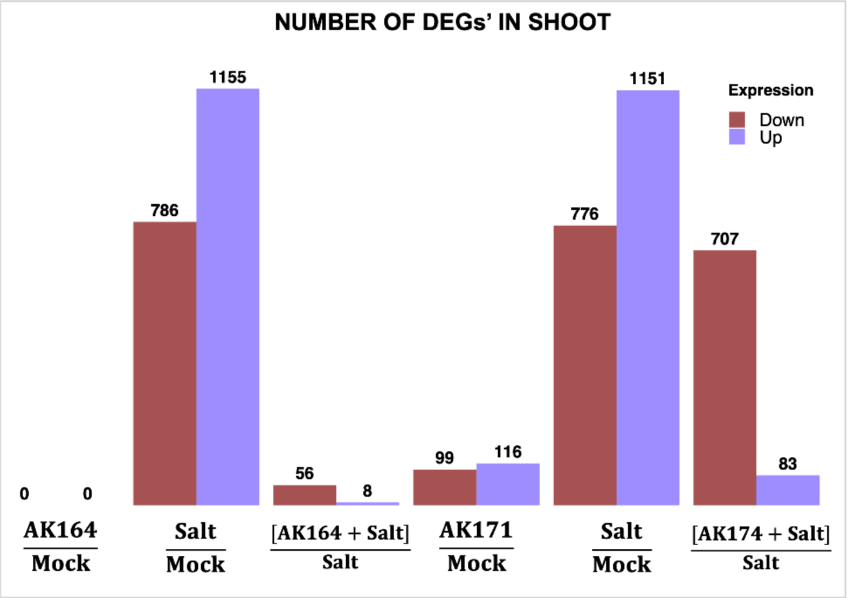

Supplementary Table 1: Beneficial impact of the best-performing mangrove isolates on the shoot and root systems of *O. sativa* seedlings (28 days old) under normal hydroponic conditions (Hoagland nutrient solution). AK031, B. seohaeanensis; AK073, Thalassospira tepidiphila; AK116, H. locisalis; AK157, Demequina activiva, AK164, I. chiayiensis; AK171, T. mobilis; AK181, P. azotoformans; AK255, I. chiayiensis; and AK266, Paenibacillus lautus. Different letters indicate statistically significant differences depending on Dunn's multiple comparison test (P < 0.05). Identical letters indicate no significant differences at P = 0.05.

		**SHOOT FRESH WEIGHT (GM%)**		**LENGTH (CM%)**		**REPRODUCTION**
NO.	***Strain***	***Shoot***	***Root***	***Shoot***	***Root***	***NO. OF TILLERS***
1	**AK031**	8.98^{ab}	5.18^{bc}	22.88^{a}	9.60^{b}	**48.13^{BC}**
2	**AK073**	25.48^{ab}	70.76^{a}	20.71^{a}	47.40^{a}	**40.76^{CD}**
3	**AK116**	28.85^{ab}	71.42^{a}	4.88^{b}	6.19^{b}	**40.76^{CD}**
4	**AK157**	31.04^{ab}	71.93^{a}	4.66^{b}	7.04^{b}	**100.00^{A}**
5	**AK164**	37.59^{a}	80.38^{a}	3.19^{b}	15.81^{ab}	**96.31^{A}**
6	**AK171**	40.49^{a}	73.65^{a}	4.88^{b}	14.17^{b}	**88.89^{AB}**
7	**AK181**	44.32^{a}	80.55^{a}	11.59^{ab}	26.05^{ab}	**77.78^{ABC}**
8	**AK255**	**31.14^{AB}**	**51.61^{AB}**	**21.68A**	**27.90^{AB}**	**74.09^{ABC}**

Supplementary Table 2: Beneficial impact of the best-performing mangrove isolates on the shoot and root systems of O. sativa seedlings (28 days old) under saline hydroponic conditions (Hoagland solution with 100mM NaCl). AK031, B. seohaeanensis; AK073, T. tepidiphila; AK116, H. locisalis; AK157, D. activiva, AK164, I. chiayiensis; AK171, T. mobilis; AK181, P. azotoformans; AK255, I. chiayiensis; and AK266, Paenibacillus lautus. Different letters indicate statistically significant differences depending on Dunn's multiple comparison test (P < 0.05). Identical letters indicate no significant differences at P = 0.05.

		**Shoot Fresh Weight (mg%)**		**Length (cm%)**		**Reproduction**
No.	***Strain***	***Shoot***	***Root***	***Shoot***	***Root***	***No. of Tillers***
1	**AK031**	60.73^{bc}	103.01^{cd}	0.49^{ab}	-3.81^{e}	3.73^{b}
2	**AK073**	53.24^{bcd}	53.25^{de}	28.46^{ab}	10.08^{bc}	-3.73^{b}
3	**AK116**	52.42^{bcd}	41.88^{de}	29.61^{ab}	21.59^{a}	-3.73^{b}
4	**AK157**	24.57^{cd}	11.91^{e}	10.85^{c}	-2.70^{de}	0.00^{b}
5	**AK164**	87.12^{ab}	284.29^{a}	38.91^{a}	20.32^{ab}	48.18^{a}
6	**AK171**	116.80^{a}	193.58^{b}	37.18^{ab}	21.35^{a}	33.33^{ab}
7	**AK181**	33.61^{cd}	45.33^{de}	12.70^{c}	6.98^{cd}	7.38^{b}
8	**AK255**	39.27^{cd}	144.71^{bc}	13.86^{c}	-18.57^{f}	14.84^{ab}

Supplementary Table 3: Beneficial increase (%) of various traits in AK164, AK171, and BiCom (AK164+AK171) colonized *O. sativa* in hydroponic growth (0 or 100 mM NaCl) compared to non-inoculated plants. Different letters indicate statistically significant differences depending on Dunn's multiple comparison test (P < 0.05). Identical letters indicate no significant differences at $p = 0.05$.

	Trait	*AK164*	*AK171*	*AK164 + AK171*
0 mM NaCl	Shoot dry weight (gm)	42.6b	49.8a	63.8a
	Root dry weight (gm)	64.2b	112.9a	118.6a
	Shoot length (cm)	10.9b	7.5a	19.2a
	Root length (cm)	22.2b	24.7a	29.1a
	No. of tillers	35.2b	82.3a	97.04a
100 mM NaCl	Shoot dry weight (gm)	101.5a	105.7a	120.3a
	Root dry weight (gm)	92.9a	90.8a	110.7a
	Shoot length (cm)	25.2b	27.7b	30.8a
	Root length (cm)	29.6b	45.6a	56.8a
	No. of tillers	54.2b	66.7a	79.2a

Supplementary Table 4: Beneficial increase (%) of various traits in AK164, AK171, and BiCom (AK164+AK171) colonized *O. sativa* growing in soil under greenhouse conditions compared to non-inoculated plants. Different letters indicate statistically significant differences depending on Dunn's multiple comparison test (P < 0.05). Identical letters indicate no significant differences at P = 0.05.

	Trait	*AK164*	*AK171*	*AK164+AK171*
0 mM NaCl	Shoot length (cm)	9a	0.4b	1.6b
	Shoot dry weight (gm)	ns	ns	10.7a
	Root dry weight (gm)	39ab	21ab	61a
	Tillers (no.)	66a	31ab	55b
	Panicles/plant (no.)	57b	43b	104a
	Spikelet/plant (no.)	15a	24b	26ab
	Grains/plant (no.)	48ab	33b	69a
	Grains/plant (gm)	29ab	25bc	54a
100 mM NaCl	Shoot length (cm)	64b	25c	92b
	Shoot dry weight (gm)	17b	26b	53a
	Root dry weight (gm)	119bc	270b	486a
	Tillers (no.)	65a	61a	90a
	Panicles/plant (no.)	136bc	206b	550a
	Spikelet/plant (no.)	20ab	38a	40a
	Grains/plant (no.)	63bc	93b	284a
	Grains/plant (gm)	96bc	152b	324a

Supplementary Table 5: Beneficial increase (%) of various traits and yield in BiCom (AK164+AK171) colonized *O. sativa* plants growing on soil (0 or 100 mM NaCl) under greenhouse conditions compared to non-inoculated plants.

	Trait	Beneficial Increase %
0 mM NaCl	Shoot length (cm)	-
	Tillers (no.)	59****
	Shoot dry weight (g)	35**
	Root dry weight (g)	41**
	Panicles/plant (no.)	37*
	Panicles/plant (gm)	31*
	Grains/plant (no.)	64*
	Grains/plant (gm)	58*
100 mM NaCl	Shoot length (cm)	3.7
	Tillers (no.)	41**
	Shoot dry weight (g)	27*
	Root dry weight (g)	198****
	Panicles/plant (no.)	1032
	Panicles/plant (gm)	4915
	Grains/plant (no.)	928
	Grains/plant (gm)	3044

*p>0.05, **P>0.01, *** P>0.001, **** P>0.0001
